# Genetic mapping of the *LOBED LEAF 1 (ClLL1)* gene to a 127.6-kb region in watermelon (*Citrullus lanatus* L.)

**DOI:** 10.1371/journal.pone.0180741

**Published:** 2017-07-13

**Authors:** Chunhua Wei, Xiner Chen, Zhongyuan Wang, Qiyan Liu, Hao Li, Yong Zhang, Jianxiang Ma, Jianqiang Yang, Xian Zhang

**Affiliations:** College of Horticulture, Northwest A&F University, Yangling, China; Universidad Miguel Hernández de Elche, SPAIN

## Abstract

The lobed leaf character is a unique morphologic trait in crops, featuring many potential advantages for agricultural productivity. Although the majority of watermelon varieties feature lobed leaves, the genetic factors responsible for lobed leaf formation remain elusive. The F_2:3_ leaf shape segregating population offers the opportunity to study the underlying mechanism of lobed leaf formation in watermelon. Genetic analysis revealed that a single dominant allele (designated *ClLL1*) controlled the lobed leaf trait. A large-sized F_3:4_ population derived from F_2:3_ individuals was used to map *ClLL1*. A total of 5,966 reliable SNPs and indels were identified genome-wide via a combination of BSA and RNA-seq. Using the validated SNP and indel markers, the location of *ClLL1* was narrowed down to a 127.6-kb region between markers W08314 and W07061, containing 23 putative ORFs. Expression analysis via qRT-PCR revealed differential expression patterns (fold-changes above 2-fold or below 0.5-fold) of three ORFs (*ORF3*, *ORF11*, and *ORF18*) between lobed and non-lobed leaf plants. Based on gene annotation and expression analysis, *ORF18* (encoding an uncharacterized protein) and *ORF22* (encoding a homeobox-leucine zipper-like protein) were considered as most likely candidate genes. Furthermore, sequence analysis revealed no polymorphisms in cDNA sequences of *ORF18*; however, two notable deletions were identified in *ORF22*. This study is the first report to map a leaf shape gene in watermelon and will facilitate cloning and functional characterization of *ClLL1* in future studies.

## Introduction

Leaves are vitally important photosynthetic organs of flowering plants, determining the distribution of nutrients, gas exchange, and water transport. Leaves furthermore exhibit a remarkable variety in size, shape, and position on the stem [1–3]. Leaf shapes reveal a clearly visible diversity among different species and even within the same species [4–8]. In addition to molecular genetic regulators, leaf shapes can also be influenced by various environmental factors, such as severe fluctuations in temperature and light regimens [9,10]. Leaf margin is an important trait of leaf shape and can be serrated, lobed, or entire (the latter phenotype is named non-lobed throughout this study) [3,11]. In general, the leaf shape character can be easily identified at the seedling stage and thus, it can be used as an efficient morphological marker to distinguish hybrids from parental lines, consequently ensuring the purity of hybrid seeds [3,12].

Leaf shape morphogenesis is a complex process and several genes have been identified that mediate its development [13]. In *Cardamine hirsuta*, the class I KNOTTED1-like homeobox (KNOX) proteins organize auxin maxima via the PINFORMED1 (PIN1) auxin efflux transporter, thus promoting leaflet initiation [14]; the transcriptional level of *KNOX* can be repressed by *CLAUSA* and *TRIPINNATE* gene products, thus affecting the leaflet number in tomato [15,16]. In Arabidopsis, the transcription factor *CUP-SHAPED COTYLEDON* rendered the genes *CUC1*, *CUC2*, and *CUC3* redundant, but showed partially distinct functions in embryonic shoot meristem formation and cotyledon boundary specification [17]; gene *CUC2* can be targeted by miR164A, and the balance between *CUC2* and miR164A determines the extent of leaf serration [18]. Loss-of-function mutations of the gene *Mt-AGO7/LOBED LEAFLET1* result in lobed leaf margins in *Medicago truncatula* [19]. The hormone cytokinin (CK) has been reported to mediate the activity of KNOX1 proteins that aid the regulation of leaf shape in tomato [20]. The homeodomain leucine-zipper transcription factor *LMI1* (*AT5G03790*) is a meristem identity regulator that interacts with LFY to activate *CAL* expression, providing additional functions in the formation of simple serrated leaves and in suppressing bract formation [21]. A previous study has demonstrated that the gene *RCO* (*REDUCED COMPLEXITY*, a *LMI1-like* homologue) is also required for leaflet development in *C*. *hirsuta* [22].

To date, several lobed leaf genes have been genetically analyzed and mapped within different species. In rapeseed (*Brassica napus* L), the *LOBED-LEAF 1* (*BnLL1*) gene has been mapped to a 36.7-kb region [3]. The major leaf shape gene (*L*) in cotton (*Gossypium hirsutum* L.) has been mapped to a 5.4 cM interval at the distal region of the short-arm chromosome 15 and two *LMI1-like* genes have been identified as the most likely candidate genes [6]. A previous study reported the HD-zip transcription factor *GhLMI1-D1b* (*Gorai*.*002G244000*) to be responsible for the majority of leaf shapes in cotton [23]. The semi-dominant allele *lma* has been mapped to a 376-kb syntenic region on chromosome 3 in the mungbean (*Vigna radiata* L.) [8]. With the use of a combination of bulk segregant analysis (BSA) and next-generation sequencing, eight QTL traits have been detected in the *Mimulus guttatus* species complex [24]. In the Cucurbitaceae family, three lobed leaf genes have been reported. For example, the lobed leaf phenotype is controlled by a recessive gene in *Cucurbita maxima* (designated *lo-1*), while in *Cucurbita ecuadorensis*, the dominant allele *Lo-2* controls the phenotype [25,26]. In melon (*Cucumis melo* L.), a single recessive gene (*pll*) contributes to the palmately lobed leaf trait and it has been located in a 14.6-kb region on CM3.5_scaffold00014 [2]. However, as far as we know, no lobed leaf gene has been cloned within any Cucurbitaceae species.

Watermelon (*Citrullus lanatus* L., 2n = 2x = 22) is a globally important cucurbit crop, accounting for 7% of the worldwide area devoted to vegetable production [27]. Currently, lobed leaf watermelon varieties dominate the market. In this study, we obtained a F_2:3_ leaf shape segregating population during the breeding process. Subsequently, we constructed a larger F_3:4_ population to fine map the lobed leaf trait in watermelon. Using a combination of BSA and the RNA-seq method, SNPs and indels were identified genome-wide. Finally, using validated SNP and indel markers, the gene *ClLL1* was mapped within a 127.6-kb interval, containing 23 putative open reading frames (ORFs). On the basis of functional annotation and qRT-PCR analysis, genes *ORF18* and *ORF22* were considered as potential *ClLL1* candidates. Further analysis revealed no polymorphisms within the cDNA sequences of *ORF18* among three watermelon genomes; however, two notable deletions were identified in *ORF22*. This study is the first to report a genetic map of the lobed leaf trait in watermelon and thus provides central information for further isolation and characterization of the gene *ClLL1*.

## Materials and methods

### Plant materials and phenotypic data collection

During the breeding process, a single plant of the commercial watermelon hybrid cultivar ‘Lingxiu’ was self-fertilized, thus obtaining F_2_ seeds. Then, a F_2_-derived F_3_ population (denoted as F_2:3_) was generated by self-pollinating F_2_ plants, which exhibited leaf shape polymorphisms (lobed and non-lobed leaves). Due to seed number limitations, the F_2:3_ plants were self-pollinated to produce a larger-sized F_3:4_ population in July 2015, which was used to map the gene *ClLL1* in this study.

The leaf shapes can clearly be distinguished after emergence of the sixth adult true leaf. Thus, to validate the inheritance pattern of the leaf shape, seeds of F_3:4_ populations were germinated and cultured in plastic greenhouses at the Northwest A&F University under natural conditions from the autumn of 2015 to the summer of 2016. The leaf phenotype of each individual was recorded after the appearance of the sixth adult leaf. Then, the data were analyzed to evaluate the segregation ratio, using the Chi-square test.

### RNA isolation and RNA-seq

A combined approach that utilized BSA and next generation sequencing of the transcriptome was applied in this study. Total RNA from young leaves of 31 and 23 lobed and non-lobed leaf seedlings at the sixth-leaf stage were extracted using the Trizol reagent (Invitrogen, Carlsbad, CA, USA) following the manufacturer’s instructions. All contaminating genomic DNA was removed via RNase-free DNaseI (TaKaRa, Dalian, China). Then, the equivalent total RNA of 31 lobed leaf and 23 non-lobed leaf individuals were pooled, constituting the lobed leaf and non-lobed leaf bulks, respectively. RNA sequencing was performed on an Illumina HiSeq™ 2000 platform and 125 bp paired-end reads were generated by Gene Denovo Co. (Guangzhou, China).

### Data processing and analysis

Using an in-house Perl program, the raw data were filtered to remove all unusable reads, which included reads that contained the Illumina library construction adapters, reads with more than 10% unknown bases, and reads with more than 50% low quality bases (Q-value ≤ 10). An index file for the watermelon reference genome “97103” (http://www.icugi.org/) was constructed with the software package Bowtie2 [27,28]. Then, high quality (HQ) clean reads were aligned to the reference genome using the software package Tophat2 with default parameters [29]. Raw SNPs and indels calling were performed via SAMtools [30]. Reliable SNPs and indels information were obtained via filtering raw sets with the following criteria: 1) mapping quality > 40; 2) read depth of the variant position > 20. All mapped reads were visually investigated using the Integrative Genomic Viewer (IGV) [31].

The differentially expressed genes (DEGs) were identified with the R package edgeR [32]. Mapped clean reads of each gene were calculated and normalized into fragments per kilobase of exon per million mapped fragments (FPKM). The false discovery rate (FDR) was used to determine the P-value threshold in multiple tests. In this study, an FDR < 0.05 and a fold change > 2 were used to judge the significance of the gene expression differences. DEGs were used for GO and KEGG enrichment analyses, similar to a method described by Zhang [33]. Both GO terms and KEGG pathways with ≤ Q-values 0.05 were significantly enriched in DEGs.

### Marker development and PCR reaction

Informed by reliable SNPs and indels, the corresponding cleaved amplified polymorphic sequence (CAPS) markers were developed and the primers were designed with Primer Premier 5 (http://www.premierbiosoft.com/).

Genomic DNA was extracted from young leaves, using the CTAB method [34,35]. Subsequent to determining the concentration of each DNA sample, approximately 200 ng genomic DNA was used as template for the PCR reaction. PCR amplification was done in a 20 μl reaction with 1 μl DNA, 10 μl PCR master mix (TSINGKE, Beijing, China), 0.5 μl of 10 μM per primer, and 8 μl distilled water. The following PCR protocol was utilized: initial denaturation at 94°C for 5 min, followed by 28 cycles at 94°C for 30 s, 57°C for 30 s, 72°C for 1 min, and a final extension at 72°C for 5 min. All PCR products were separated on 2% agarose gels. Markers exhibiting length polymorphisms were directly used for the genetic mapping, while the rest were digested with their specific endonuclease at 37°C or 65°C for 4 h, followed by analysis on agarose gel.

### Mapping strategy

For primary linkage analysis, we screened 93 F_3:4_ individuals with 11 polymorphic markers, which were designed for each chromosome according to SNPs identified from RNA-seq data. Subsequent to obtaining the linked marker, new flanking markers were designed to screen the small F_3:4_ population. After delimiting the target gene between two markers, a larger population consisting of 781 F_3:4_ individuals, was used to identify recombinants. All recombinants identified from both populations were carefully transplanted into the field and used to extract a large amount of genomic DNA. To further localize the primary interval, a series of new markers were developed based on reliable SNPs and indels (after removing the low quality data, using the criteria mentioned above). These markers were subsequently used to screen the recombinants. Markers used in the gene mapping strategy are listed in Table 1.

**Table 1:** List of all primers for 12 markers used in genetic mapping of *ClLL1*.

Marker name	Physical location	Primer sequence (5->3)	PCR product (bp)	Endonuclease	Annealing temp (°C)	Marker type
W01144	Chr04:20818124	F:ACCAAGCTACCCCAACCCACC	932	BamHI	57	CAPS
		R:TCCAAGATTGGGAGGCGGTGC				
W01211	Chr04:19907542	F:AGGCCTGAGAATGCTCTGGGGA	966	XhoI	57	CAPS
		R:TTCCTCGGGACCGACACGGAG				
W03041	Chr04:20857870	F:TAGACTGGGCGGAAGAGACGGC	640	TaqI	57	CAPS
		R:ACTAATCCATCCCCGAGCACACCT				
W03042	Chr04:20889818	F:ACCATACAGCGCTGAAACTCTGCT	664	BstNI	57	CAPS
		R:GCTTGCCTCCAGCTTCGCATGA				
W06252	Chr04:20914978	F:GCCAAACTAATCATACATACAG	920	EcoRV	57	CAPS
		R:GACATCAATAACATCCCAAGA				
W08314	Chr04:21111771	F:TTATTCTCAATAAACGCCCTTCCCTAGTA	140	EcoRV	57	CAPS
		R:CAGCGACATTTTGCAATATTTGAAGATAT				
W07061	Chr04:21239403	F:TGGTTGAGGCCGAAGAGGTTGGT	844	RsaI	57	CAPS
		R:TTATGGGCATGCAGTGTGGGGC				
W07062	Chr04:21262238	F:GATTTGGCGTTACATCTGCTT	789	TaqI	57	CAPS
		R:GTTACATTTGAGACATTTGGGT				
W07063	Chr04:21294844	F:TTTCATTTGGTCCCTATGTTT	275		57	SCAR
		R:AACCTCAATTATTAACTAACTACTCAC				
W06253	Chr04:21505058	F:AAAGGCTTGGATTATGGAATT	753	PstI	57	CAPS
		R:AAAAGTTGTGGGTTAGGGAAT				
W0130	Chr04:22250767	F:GCCCTGGCCGGACACAGGATA	535	XagI	57	CAPS
		R:CGACGTCGTCAAAGCCAGCATC				
W01214	Chr04:23498056	F:TGGGCTCAAGACATAGAACATGCCA	538	HindIII	57	CAPS
		R:AGCTCATCCCACCGATTCATGTGT				

### Gene prediction and qTR-PCR

The predicted genes in the mapping interval were downloaded from the watermelon database (http://www.icugi.org/) [27]. The genomic sequence of the mapping interval was extracted from the watermelon reference genome using an in-house Perl program. The candidate gene prediction was further performed using FGENESH (http://linux1.softberry.com/). The function of thus predicted genes was retrieved from NCBI using the BLASTp tool (https://blast.ncbi.nlm.nih.gov/Blast.cgi).

Quantitative real-time PCR (qRT-PCR) was performed to identify all candidate genes. Gene-specific primers were designed based on the open reading frame sequence of predicted genes, using the software Primer Premier 5 with an annealing temperature range of 57–60°C and an amplification length between 200 and 300 bp. The housekeeping gene *Actin* was used as internal control [36]. The first-strand cDNA was synthesized using a FastQuant RT kit (TIANGEN, Beijing, China) and qRT-PCR was performed on a StepOnePlus Real-Time PCR platform (Applied Biosystems, Foster City, CA, USA). The values from reactions in triplicate were analyzed with the 2^-^ΔΔ^Ct^ method [37]. SPSS 21.0 software was used for statistical analysis, and the data are presented as mean values ± SD. The differential expression significance between lobed and non-lobed leaf individuals was verified via Student’s t-test. All primer pairs used in the qRT-PCR experiment are listed in S1 Table.

### Sequence analysis

Total RNA was extracted from leaves of both lobed and non-lobed leave plantlets, using the TriZol reagent (Invitrogen, Carlsbad, CA, USA) and first-strand cDNA was synthesized using a FastQuant RT kit (TIANGEN, Beijing, China). To amplify full-length sequences of candidate genes, gene-specific primers were designed using the software Primer Premier 5 and the results are listed in S1 Table. Nested PCR was performed to amplify the cDNA sequence of *ORF22*. PCR amplification was conducted in a 25 μl reaction vessel with one unit of Fast Pfu Taq (TransGen, Beijing, China), treated for 4 min at 95°C, followed by 30 cycles at 95°C for 30 s, 58 or 60°C for 20 s, and a final extension at 72°C for 2 min. The PCR products were purified and ligated into the vector pEASY-T1, using the TA clone kit (TransGen, Beijing, China). At least three positive clones per sample were sent for sequencing. The cDNA sequences of candidate genes *ORF18* (*Cla018357*) and *ORF22* (*Cla018360*) were downloaded from the watermelon genome database “97103” (http://www.icugi.org/). Sequence analysis was performed using the software Geneious (http://www.geneious.com).

## Results

### Genetic analysis of the lobed leaf trait

**Fig 1:**
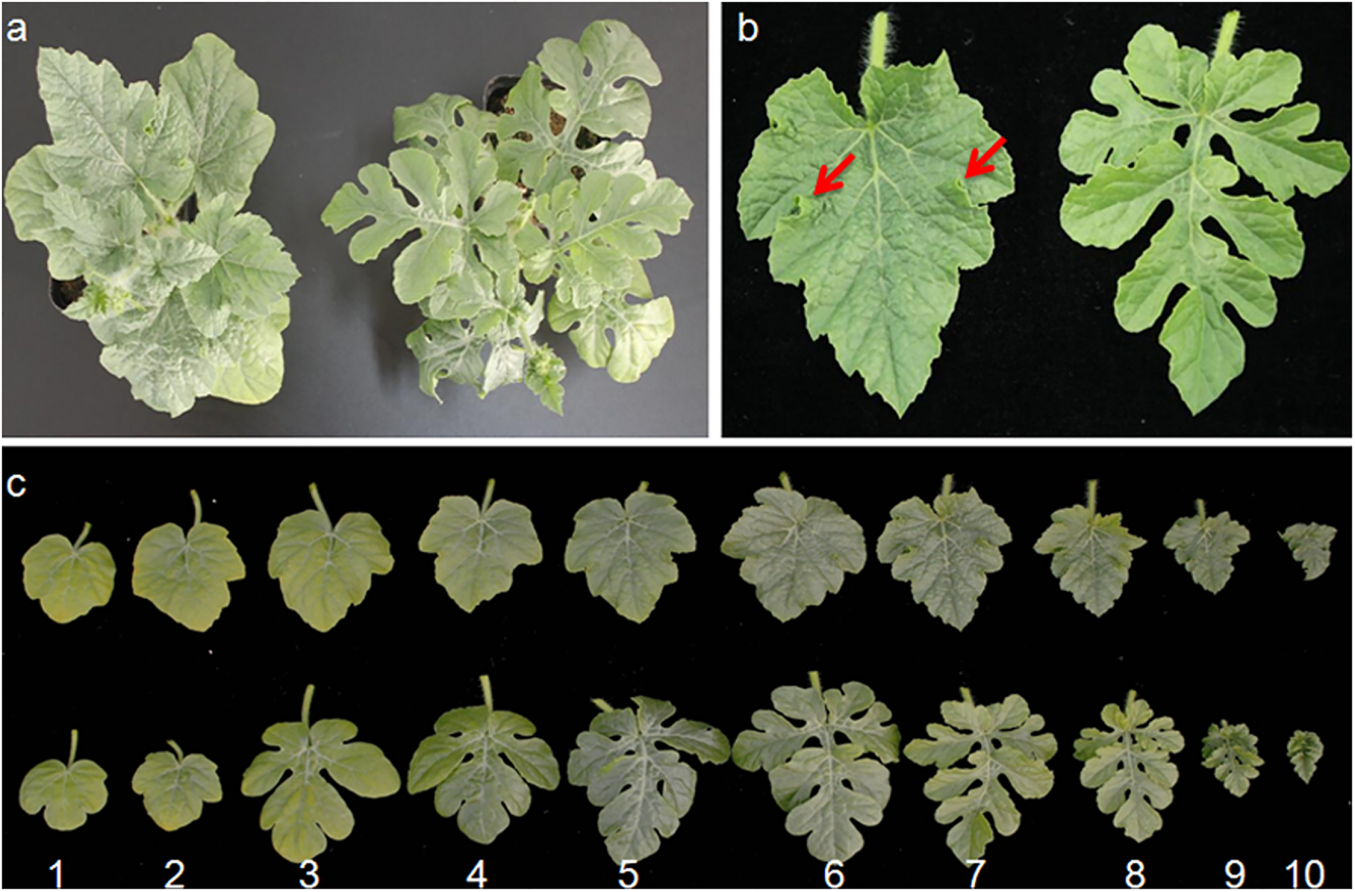
Phenotypes of leaf shapes in watermelon. (**a)** Lobed and non-lobed leaf watermelon seedlings. (**b)** Two different leaf shapes in watermelon. Small symmetrical marginal protrusions are marked with red arrows. (**c)** Comparison of the first ten true leaves from lobed and non-lobed phenotype seedlings.

Individuals from a F_2:3_ population derived from the watermelon cultivar ‘Lingxiu’ during the breeding process, showed leaf form polymorphisms (lobed and non-lobed leaves). However, due to seed number limitation, it was impossible to perform the genetic mapping of the lobed leaf gene with this population. Therefore, the F_2:3_ plants were self-pollinated to produce a sufficiently sized F_3:4_ population. Consistent with F_2:3_ plants, the leaves of F_3:4_ individuals were classified as either lobed or non-lobed (Fig 1A). Moreover, the margins of non-lobed leaves were not smooth and featured slight serrations and small symmetrical marginal protrusions (Fig 1B). Therefore, to further observe the phenotypic variations, the first ten true leaves from both lobed and non-lobed seedlings were compared. As shown in Fig 1C, the leaf shapes can be visibly distinguished after the emergence of the third true leaf and differentiated at the sixth adult leaf stage. In addition, the symmetrical marginal protrusions mentioned above can clearly be observed on the sixth true leaf from non-lobed leaf plants. A small proportion of F_3:4_ progenies was planted to analyze the genetic inheritance of the lobed leaf trait. As a result, 93 F_3:4_ individuals presented two phenotypes, including 69 lobed and 24 non-lobed plants, with a 3:1 Mendelian ratio (χ^2^ = 0.03, p = 0.86). Furthermore, leaves of seedlings derived from non-lobed F_2:3_ individuals had a leaf form consistent with the parent plant. In summary, we inferred that a dominant allele, designated as *ClLL1*, controlled the lobed leaf trait in watermelon.

### Genome-wide identification of SNPs and indels

To identify the SNPs and indels genome-wide, a method combining BSA and RNA-seq was used in this study. After removing low-quality sequences from the raw data, a total of 53 and 50 million clean reads were generated from lobed and non-lobed bulks, with approximately 6.59 and 6.19 Gbp data, respectively (Table 2). The sequencing data has been submitted to NCBI and can be accessed in the respective short read archives (SRR5100272 and SRR5100273). Using strict criteria to filter the clean data, high quality (HQ) reads that accounted for 96% of the clean data were generated per bulk. The Q30 values of two samples were 89.02% and 88.31%, respectively. Then, these HQ clean reads were mapped to the watermelon reference genome using the software Bowtie2 and Tophat2 [28,29]. As a result, approximately 85% of all reads could be mapped onto chromosomes and a total of 16,848 SNPs and indels were identified. Then, after removing the low quality sites (read counts < 20; quality < 40), 5,966 reliable SNPs and indels were selected and utilized to develop markers in the further analysis (Table 3 and S2 Table). The distribution of SNPs and indels on chromosomes showed considerable variation. E.g., chromosome 6 had the largest number (948) of SNPs and indels, while only 218 were located on chromosome 4.

**Table 2:** Detailed information of RNA-seq data of lobed and non-lobed leaf bulks.

	Lobed Leaf	Non-lobed Leaf
**Clean Reads Num**	52698774	49553160
**Clean Data(bp)**	6587346750	6194145000
**Q30 Percentage**	87.87%	86.77%
**HQ Clean Reads Num**	51015830	47474006
**HQ Clean Data(bp)**	6376978750	5934250750
**Q30 Percentage**	89.02%	88.31%
**Unique Mapped Reads**	43340214 (84.95%)	39878758 (84.00%)
**Multiple Mapped reads**	515006 (1.01%)	455174 (0.96%)
**Unmapped Reads**	7134152 (13.98%)	7108416 (14.97%)

**Table 3:** Distribution of reliable SNPs and indels on watermelon chromosomes.

	Chr0	Chr1	Chr2	Chr3	Chr4	Chr5	Chr6	Chr7	Chr8	Chr9	Chr10	Chr11
**SNP**	77	575	363	295	197	670	881	490	334	573	435	463
**Indels**												
Insertion	3	30	28	20	14	39	35	28	31	31	29	27
Deletion	1	28	34	22	7	32	32	34	27	31	26	24
**Total**	81	633	425	337	218	741	948	552	392	635	490	514

Chr0 represents unanchored supper-scaffolds

### Identification of DEGs

The total mapped reads were used to analyze the DEGs with the criteria of FDR < 0.05 and fold change > 2. As a result, a total of 195 DEGs were identified within both bulked pools; among those, 133 were up-regulated and 62 were down-regulated (see S3 Table). Functional annotations of the DEGs, as well as the GO and KEGG enrichment analyses, revealed that a large proportion of transcription genes (such as the HD-zip transcription factor *Cla014193*) may be involved in watermelon leaf-shape morphogenesis.

### Primary mapping of *ClLL1*

**Fig 2:**
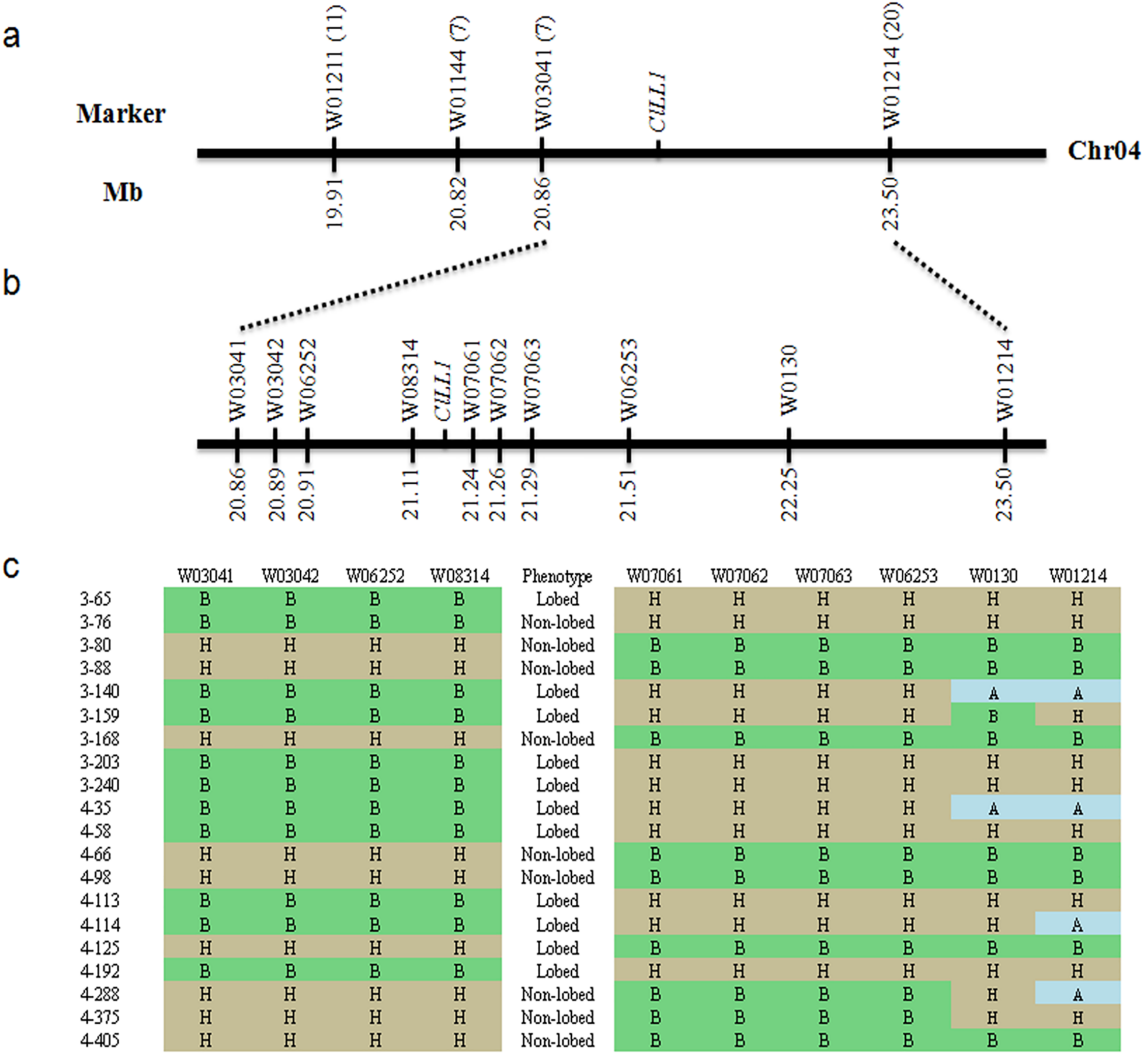
Genetic mapping of the lobed leaf trait gene *ClLL1* in watermelon. (**a)** Primary mapping of *ClLL1* using 93 F_3:4_ individuals. Gene *ClLL1* was delimited to the region between markers W03041 and W01214. The numbers in brackets after the marker name indicate the numbers of recombinants. (**b)** Fine mapping of *ClLL1*. The gene *ClLL1* was fine mapped in a l27.6-kb region between markers W08314 and W07061. (**c**) Marker genotypes of the recombinants near the lobed leaf gene *ClLL1* between W08314 and W07061. The alleles are abbreviated according to their origin: A: Lobed leaf; B: Non-lobed leaf; H: Heterozygous.

To locate gene *ClLL1* on a chromosome, 11 markers were designed per chromosome (data not shown) and used to screen the F_3:4_ population (93 individuals). Linkage analysis revealed that marker W01144 on chromosome 4 was linked to the lobed leaf trait. However, the polymorphic bands produced by W01144 were not easy to distinguish; therefore, a new marker W03041 was developed which is physically adjacent to W01144. Then, W03041 was used to genotype 93 mapping individuals and seven recombinants were detected with this technique (Fig 2A). To determine the primary region for gene *ClLL1*, two flanking markers, W01211 (on the left side of W03041) and W01214 (on the right side of W03041), were developed to screen the population. Subsequent linkage analysis suggested that both markers W01211 and W03041 were located on the same side of gene *ClLL1*, with 11 and 7 recombinants and at distances of 5.9 and 3.8 cM, respectively. However, marker W01214 was situated on the other side, with 20 recombinants at a 12.9 cM genetic distance from *ClLL1* (Fig 2A). Thus, we concluded gene *ClLL1* to be roughly delimited within a 2.64 Mb region between markers W03041 and W01214, with a 16.7 cM genetic distance.

### Fine mapping of *ClLL1*

To precisely identify the genomic region surrounding gene *ClLL1*, a larger segregating population consisting of 781 individuals was utilized, which segregated 596 lobed and 185 non-lobed phenotypes, fitting a 3:1 ratio (χ^2^ = 0.72, p = 0.40). The primary flanking markers W03041 and W01214 were utilized to screen this population and a further new 39 and 146 recombinants were identified. Combined with the recombinants obtained in the first population, a total of 212 recombinants were obtained. Consequently, seven new markers were designed for the primary region (Fig 2B), and were used to phenotype 212 recombinant individuals. Finally, gene *ClLL1* was delimited to be between markers W08314 and W07061, with 18 and 2 recombinants and at genetic distances of 1.15 and 0.13 cM, respectively (Fig 2B and 2C). Since we had no reliable SNPs or indels to develop new polymorphic markers, further localization of this mapping region was unfeasible. Based on the genetic distance between gene *ClLL1* and both flanking markers, we inferred the location of *ClLL1* to be closer to W07061. The physical distance between W08314 and W07061 was approximately 127.6-kb, according to the watermelon reference genome sequence.

### Candidate genes for *ClLL1*

According to the watermelon genome annotation database (http://www.icugi.org/), 14 putative genes (*Cla018348* to *Cla018361*) were annotated in the candidate region (Table 4). Gene prediction was further conducted via FGENESH (http://softberry.com), yielding nine additional putative ORFs. Then, all 23 putative amino acid sequences were subjected to BLASTP (NCBI), revealing that seven out of these nine ORFs (*ORF2*, *4*, *8*, *12*, *15*, *17*, and *21*) identified by FGENESH possessed no effective annotations (E-value cutoff of 1e^-10^), and were consequently discarded for future analysis. *ORF5* (*Cla018350*), *ORF9*, and *ORF18* (*Cla018357*) encode unknown proteins (Table 4). The *ORF1* (*Cla018348*) protein exhibited 89% similarity with a predicted aspartic proteinase-like protein. *ORF3* (*Cla018349*), *ORF6* (*Cla018351*), *ORF7* (*Cla018352*), and *ORF10* (*Cla018353*) are homologues, sharing at least 55% amino acid sequence identity and encoding a putative threonine dehydratase. *ORF11* (*Cla018354*) encodes a GDSL-motif lipase/hydrolase family protein. The remaining genes encode a pentatricopeptide repeat protein (*ORF13*), beta-galactosidase-like protein (*ORF14*), glycosyltransferase (*ORF16*), serine/threonine protein kinase (*ORF19*), 60S ribosomal protein L24 (*ORF20*), homeobox-leucine zipper-like protein (*ORF22*), and pyruvate kinase (*ORF23*).

**Table 4:** Predicted genes between markers W08314 and W07061.

ORF. no	Position	CDS	Gene id	NCBI BlastP Hit
*ORF1*	Chr4:21115062..21120010	1567	*Cla018348*	Aspartyl protease-like protein
*ORF2*	Chr4:21126988..21129534	51		No annotation
*ORF3*	Chr4:21130256..21133784	1845	*Cla018349*	Threonine dehydratase
*ORF4*	Chr4:21135471..21139884	150		No annotation
*ORF5*	Chr4:21142412..21142771	359	*Cla018350*	Unknown Protein
*ORF6*	Chr4:21143742..21147413	1414	*Cla018351*	Threonine dehydratase
*ORF7*	Chr4:21156327..21160590	1839	*Cla018352*	Threonine dehydratase
*ORF8*	Chr4:21161184..21165631	198		No annotation
*ORF9*	Chr4:21166844..21167799	267		uncharacterized protein
*ORF10*	Chr4:21171749..21176612	1887	*Cla018353*	Threonine dehydratase
*ORF11*	Chr4:21178315..21180439	1051	*Cla018354*	GDSL-motif lipase/hydrolase family protein
*ORF12*	Chr4:21181007..21182362	312		No annotation
*ORF13*	Chr4:21183583..21186210	2627	*Cla018355*	Pentatricopeptide repeat protein
*ORF14*	Chr4:21187818..21189921	480		Beta-galactosidase-like
*ORF15*	Chr4:21191643..21193351	87		No annotation
*ORF16*	Chr4:21194156..21197263	1585	*Cla018356*	Glycosyltransferase
*ORF17*	Chr4:21200223..21205764	165		No annotation
*ORF18*	Chr4:21208684..21212333	1357	*Cla018357*	uncharacterized protein
*ORF19*	Chr4:21213450..21215972	1637	*Cla018358*	Serine/threonine protein kinase
*ORF20*	Chr4:21220810..21222360	490	*Cla018359*	60S ribosomal protein L24
*ORF21*	Chr4:21223829..21231305	123		No annotation
*ORF22*	Chr4:21232016..21234402	699	*Cla018360*	Homeobox-leucine zipper-like protein
*ORF23*	Chr4:21242282..21257426	1568	*Cla018361*	Pyruvate kinase

**Fig 3:**
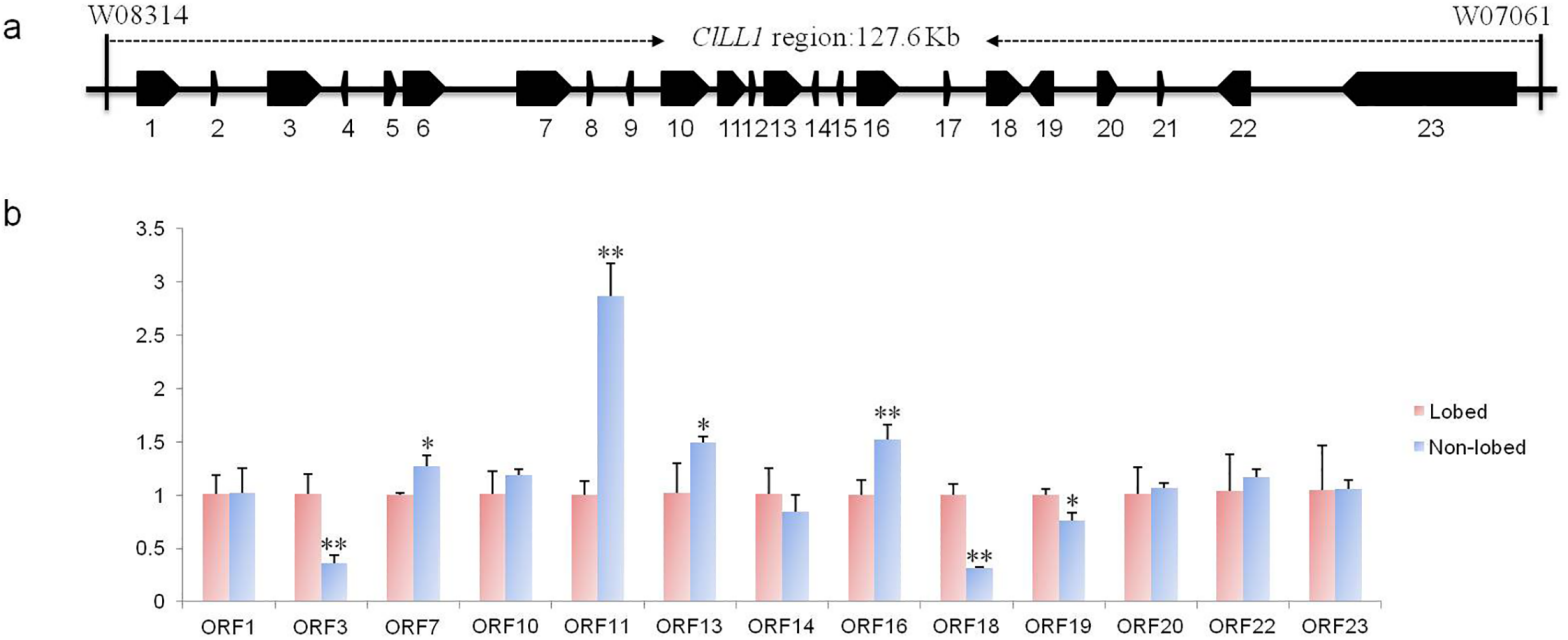
Prediction and relative expression level of candidate genes in the *ClLL1* region. (**a)** 23 putative ORFs were predicted in a 127.6-kb region between makers W08314 and W07061. (**b)** The relative expression level of candidate genes in both lobed and non-lobed leaf plants. The data are presented as average values of three replicates (mean value ± SD). “*, **” represent significant differences at *p* < 0.05 and *p* < 0.01, respectively, according to the Student’s t-test. *Actin* was used as an internal control.

To further determine possible candidates, we designed specific primers associated with 16 predicted genes (except for *ORF2*, *ORF4*, *ORF8*, *ORF12*, *ORF15*, *ORF17*, and *ORF21*) in the 127.6-kb region, and analyzed their expression levels in both lobed and non-lobed plants (Fig 3A). The results indicated that the transcription of *ORF5*, *ORF6*, and *ORF9* could not be detected. *ORF1*, *ORF10*, *ORF14*, *ORF20*, *ORF22*, and *ORF23* revealed no expression differences between lobed and non-lobed plants (Fig 3B). Using fold-changes above 2-fold or below 0.5-fold as thresholds, only *ORF11* was expressed at a significantly higher level (~2.9 fold) in non-lobed leaf plants compared to lobed leaf plants. *ORF11* was homologous to gene *AT5G03820* in Arabidopsis, which belongs to the GDSL-motif lipase/hydrolase family and may be involved in seed morphology [38]. The expression level of two genes (*ORF3* and *ORF18*) was repressed in non-lobed plants, with 0.36 and 0.31 fold changes compared to lobed plants. *ORF3* encodes a threonine dehydratase protein, which is homologous to protein OMR1, catalysing the deamination and dehydration of threonine [39]. *ORF18* encodes an uncharacterized protein with unknown function. It is worth to note that *ORF22* with identical expression levels in both samples encodes a homeobox-leucine zipper-like protein (Fig 3B and Table 4). It is a homologue of the gene *LMI1* (*AT5G03790*), which has been reported to interact with LFY and CAL, functioning as a meristem identity regulator [3,21]. A previous study confirmed that gene *RCO* (homologous to *LMI1*) was required for leaflet development in *C*. *hirsuta* [22]. Moreover, *LMI1* homologues *Bra009510* in rapeseed as well as *Gorai*.*002G244000* in cotton were identified as most likely candidate genes for leaf shape loci in these species [3,6,23]. In summary, according to gene annotation and expression analysis, as well as the genetic distance between gene *ClLL1* and two flanking markers, we inferred that both *ORF18* (encoding an unknown protein) and *ORF22* (homologous to *LMI1*) are two possible candidate genes for the lobed leaf shape trait of watermelon.

**Fig 4:**
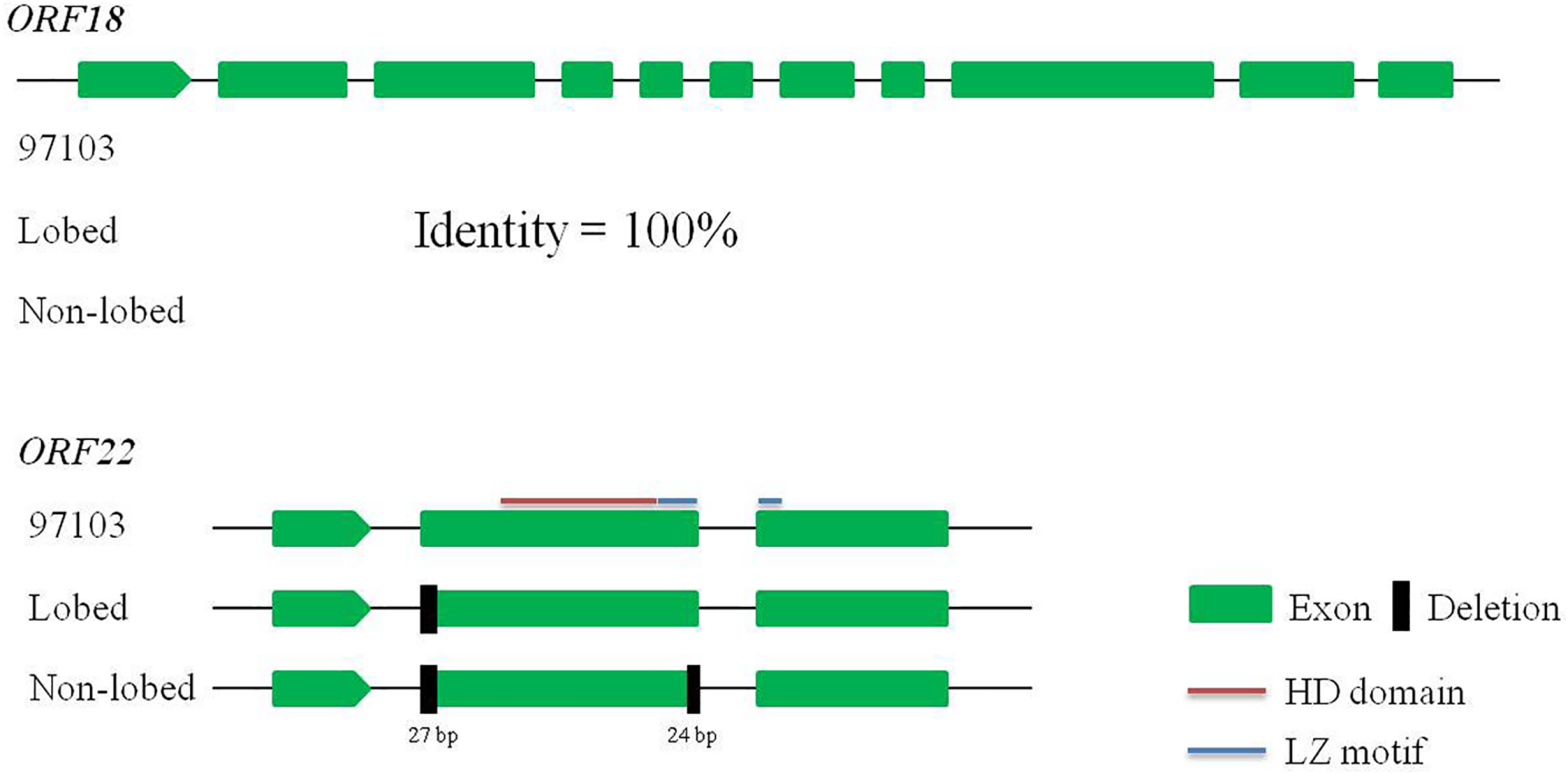
Nucleotide polymorphisms of *ORF18* and *ORF22* among three watermelon genomes. No nucleotide polymorphisms were identified in the cDNA sequences of *ORF18* among three genomes. Two deletions (27-bp and 24-bp) were found in the second exon of *ORF22* among three genomes. The homeodomain (HD) domain and leucine zipper (LZ) motif of *ORF22* were predicted by the software Pfam (http://pfam.xfam.org/).

To verify this assumption, we analyzed the nucleotide sequence polymorphisms of the two possible candidate genes. Based on the RNA-seq data, the coverage of mapped reads on these predicted genes was visually investigated, using the Integrative Genomics Viewer (IGV) software (S1 Fig). As a result, only the 14 ORFs annotated in the watermelon genome database were covered by sequenced reads. However, no reliable SNPs or indels were detected in these predicted genes. It is worth to note that *ORF5*, *ORF6*, and *ORF22* had only few mapped reads, which may affect the identification of nucleotide polymorphisms in these genes. To further identify DNA polymorphisms among leaf shapes, we designed gene specific primers for the two candidate genes (*ORF18* and *ORF22*) and amplified full-length cDNA sequences from lobed and non-lobed leaf individuals. Additionally, cDNA sequences of both *ORF18* and *ORF22* were downloaded from the reference genome database of the watermelon cultivar “97103”. Sequence alignment with *ORF18* showed no nucleotide polymorphisms among three genomes (Fig 4). Sequence analysis of *ORF22* identified two prominent polymorphisms among three genomes. First, a 27-bp deletion (encoding nine amino acids) was found to be located at the start of the second exon in both lobed and non-lobed leaf genomes. The second notable polymorphism was a 24-bp deletion (encoding eight amino acids) at the end of the second exon was found only in the non-lobed leaf genome (Fig 4). It is worth to note that neither of these two deletions resulted in a translation frameshift (S2 Fig). However, the 24-bp deletion that encoded eight amino acids in the leucine zipper (LZ) motif may disturb the characteristic spacing of the leucine zipper and interfere with gene function.

## Discussion

The watermelon is an important cucurbit crop planted widely throughout the world [27]. Currently, lobed leaf shape watermelon varieties dominate the market. However, both genetic control and underlying mechanisms that lead to the formation of lobed leaf shape in watermelon are still poorly understood. In this study, we revealed that the lobed leaf trait in watermelon was controlled by a single dominant allele, named *ClLL1*. Environmental factors, such as strong fluctuations in temperature or light regimens, were reported to adversely affect both leaf growth processes and leaf shape [10]. For example, the palmately lobed leaf trait in melon, another important cucurbit crop, was controlled by the single recessive gene *pll*, which is only expressed in individuals grown in the field [2]. However, the lobed leaf phenotype in watermelon can steadily be observed after the emergence of the sixth true leaf in plants either grown in the field or in artificial climate chambers and greenhouse conditions, indicating that the expression of gene *ClLL1* is not or only slightly affected by environmental factors. It has been reported that the cucurbit genome speciation event occurred 15–23 million years ago [27]. Therefore, it will be interesting to further investigate the underlying mechanism of leaf shape formation in these two cucurbit relatives.

In the present study, a combined BSA and RNA-seq approach was utilized for genome-wide identification of SNPs and indels between two bulked pools, which has widely been used to develop molecular markers in gene mapping [40–42]. Moreover, a total of 195 DEGs were identified, including 133 up-regulated and 62 down-regulated genes (S3 Table). Using a F_3:4_ population (N = 874) and 12 molecular markers, we successfully delimited *ClLL1* to a 127.6-kb interval between markers W08314 and W07061 (Fig 2). The mapping region could not be further narrowed down, due to the limitations of reliable SNPs and indels within this interval. Please note that W08314 and W07061 located at 1.15 and 0.13 cM genetic distances from the lobed leaf trait, respectively, inferred gene *ClLL1* to be closer to W07061. Sequence annotation analysis showed that there are 23 putative ORFs in this region (Table 4). According to SNPs and indels identified from RNA-seq data, we detected no reliable nucleotide polymorphisms in these predicted genes (S1 Fig). Using qRT-PCR assays, we found that *ORF11* was expressed at a substantially higher level (more than twofold) in non-lobed leaf plants compared to lobed leaf plants, while the expression level of *ORF3* and *ORF18* had decreased (more than twofold) in the former (Fig 3). *ORF11* encoding a GDSL-motif lipase/hydrolase family protein exhibited higher amino acid similarity to *AT5G03820* in Arabidopsis. Gene *AT5G03820* has been reported to be down-regulated by the bHLH transcription factor gene *RGE1*, possibly causing disordered hormone flux in the endosperm to function in the seed morphology [38]. *ORF3* is homologous to the threonine dehydratase protein OMR1. A previous study indicated that OMR1 catalyses the deamination and dehydration of threonine, which is the first and also the committed step in the biosynthesis of isoleucine [39]. *ORF18* encodes an uncharacterized protein, with significantly repressed expression level in non-lobed leaf plants. It is worth mentioning that *ORF22* is a *LMI1* (*AT5G03790*) homologue in Arabidopsis, whose function is involved in the formation of simple serrated leaves [21]. It has been reported that the gene *RGO* (a homologue of *LMI1*) plays an important role in the leaflet development in *C*. *hirsuta* [22], and the most possible candidate genes of the lobed leaf trait in rapeseed and cotton are also characterized as *LMI1* homologues [3,6,23]. Using the software IGV and our RNA-seq data, we found that only few reads were mapped on gene *ORF22*, which may affect the detection of SNPs and indels. However, sequence alignment revealed that two notable deletions (27-bp and 24-bp) were identified in the cDNA sequences of *ORF22* among three genomes, and the second polymorphism (24-bp deletion encoding eight amino acids) in the LZ motif may disrupt the function of the allele. Therefore, based on gene annotation and sequence analysis, *ORF22* is still recognized as a candidate responsible for the lobed leaf phenotype in watermelon.

Leaves are important photosynthetic organs of plants. To survive in different habitats, plants have the ability to adapt leaf position, size, and shape [4,24]. A lobed leaf has numerous significant functional advantages in many aspects. For example, a lobed leaf has a lower hydraulic resistance, which may constitute a mechanism to improve the water balance under dry conditions [43]. The rate of heat transfer from lobed leaves is greater than in non-lobed leaves, thus reducing leaf temperature to prevent sunburn in plants [2,4]. Furthermore, leaf shapes can be used in hybrid production as visible morphological markers [3]. Therefore, an improved understanding of genes and underlying regulator networks associated with lobed leaf shape may allow us to take full advantage of leaf shape in the breeding process. In this study, we performed genetic mapping of the lobed leaf gene *ClLL1* in watermelon and identified the candidate genes *ORF18* and *ORF22*, which will shed light on the molecular mechanism of lobed leaf formation in this important cucurbit crop.

## Supporting information

S1 Table Detailed information of primers used in the qRT-PCR assays and gene cloning. (XLS)

S2 Table All reliable SNPs and indels identified via RNA-seq data of lobe and non-lobed bulks. (XLS)

S3 Table Detailed information of differentially expressed genes. (XLS)

S1 Fig Coverage of mapped reads on the predicted genes within the *ClLL1* interval. **a** 23 putative ORFs in the 127.6-kb region. **b** Mapping results of RNA-seq data of lobed and non-lobed leaf bulks. Blue lines and rectangles indicate the 14 annotated genes in the watermelon genome database. Grey rectangles indicate the mapped reads from the RNA-seq data of each bulk. The visualization of mapping reads has been exhibited in IGV software.
(TIF)

S2 Fig Amino acid mutations caused by two deletions (27-bp and 24-bp) in ORF22 among three genomes. (TIF)
