# Supplementary figures and images for: Genetic mapping of the LOBED LEAF 1 (ClLL1) gene to a 127.6-kb region in watermelon (Citrullus lanatus L.)
Source: PLoS One. 2017 Jul 13;12(7):e0180741. doi: 10.1371/journal.pone.0180741 (PMC5509165; doi:10.1371/journal.pone.0180741)

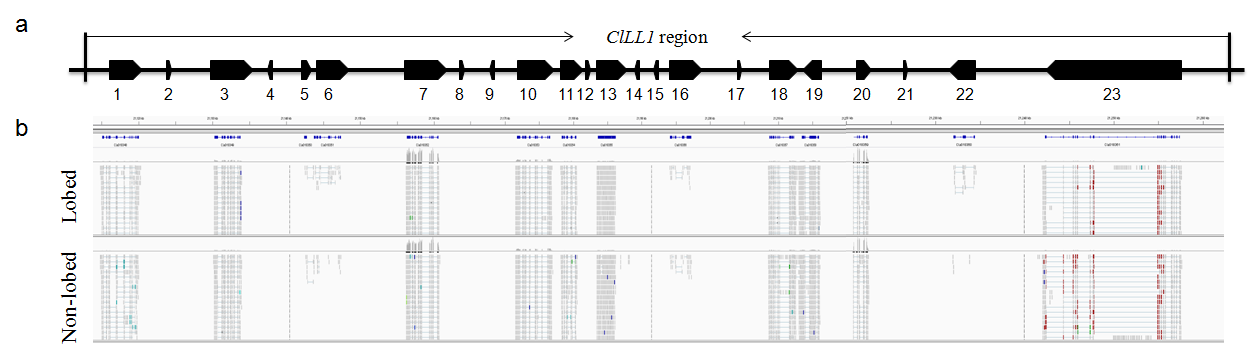

Supplement: S1 Fig — a 23 putative ORFs in the 127.6-kb region. b Mapping results of RNA-seq data of lobed and non-lobed leaf bulks. Blue lines and rectangles indicate the 14 annotated genes in the watermelon genome database. Grey rectangles indicate the mapped reads from the RNA-seq data of each bulk. The visualization of mapping reads has been exhibited in IGV software. (TIF) [file pone.0180741.s004.tif]

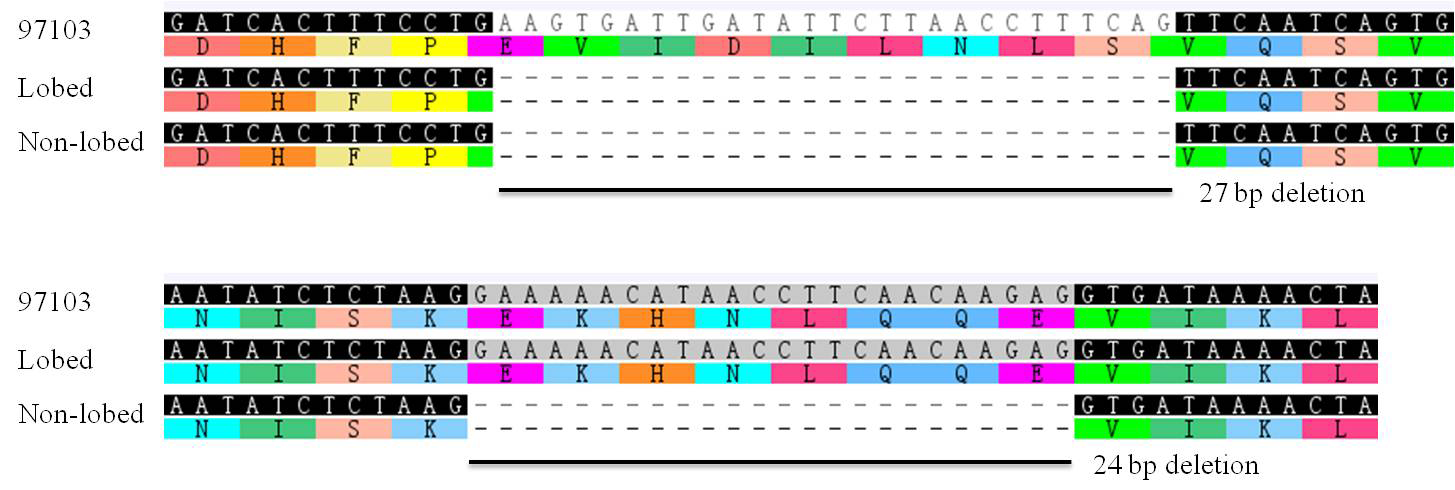

Supplement: S2 Fig — (TIF) [file pone.0180741.s005.tif]
